# Biosynthesis of the antimicrobial cyclic lipopeptides nunamycin and nunapeptin by *Pseudomonas fluorescens* strain In5 is regulated by the LuxR‐type transcriptional regulator NunF

**DOI:** 10.1002/mbo3.516

**Published:** 2017-08-06

**Authors:** Rosanna C. Hennessy, Christopher B. W. Phippen, Kristian F. Nielsen, Stefan Olsson, Peter Stougaard

**Affiliations:** ^1^ Department of Plant and Environmental Sciences University of Copenhagen Copenhagen Denmark; ^2^ Department of Systems Biology Technical University of Denmark Lyngby Denmark; ^3^ State Key Laboratory of Ecological Pest Control for Fujian and Taiwan Crops Fujian Agriculture and Forestry University Fujian China

**Keywords:** Antimicrobial activity, bacterial–fungal interactions, LuxR regulation, nonribosomal peptides, *Pseudomonas*, secondary metabolism

## Abstract

Nunamycin and nunapeptin are two antimicrobial cyclic lipopeptides (CLPs) produced by *Pseudomonas fluorescens* In5 and synthesized by nonribosomal synthetases (NRPS) located on two gene clusters designated the *nun–nup* regulon. Organization of the regulon is similar to clusters found in other CLP‐producing pseudomonads except for the border regions where putative LuxR‐type regulators are located. This study focuses on understanding the regulatory role of the LuxR‐type‐encoding gene *nunF* in CLP production of *P. fluorescens* In5. Functional analysis of *nunF* coupled with liquid chromatography–high‐resolution mass spectrometry (LC‐HRMS) showed that CLP biosynthesis is regulated by *nunF*. Quantitative real‐time PCR analysis indicated that transcription of the NRPS genes catalyzing CLP production is strongly reduced when *nunF* is mutated indicating that *nunF* is part of the *nun–nup* regulon. Swarming and biofilm formation was reduced in a *nunF* knockout mutant suggesting that these CLPs may also play a role in these phenomena as observed in other pseudomonads. Fusion of the *nunF* promoter region to mCherry showed that *nunF* is strongly upregulated in response to carbon sources indicating the presence of a fungus suggesting that environmental elicitors may also influence *nunF* expression which upon activation regulates nunamycin and nunapeptin production required for the growth inhibition of phytopathogens.

## 1 INTRODUCTION

*Pseudomonas fluorescens* strain In5 originally isolated from an agricultural suppressive soil in southern Greenland is a promising biocontrol agent capable of suppressing *Rhizoctonia solani* infection of tomato seedlings and inhibiting the growth of diverse phytopathogens (Michelsen, Watrous, Glaring, Kersten, Koyama, et al., 2015). Biocontrol agents provide a potential alternative to synthetic chemicals for crop protection against disease. The cyclic lipopeptides (CLP) nunamycin and nunapeptin are a distinctive feature of the Greenlandic strain *P. fluorescens* In5 and have recently been shown to play a key role in the suppressiveness of soilborne pathogens by the bacterium (Michelsen, Watrous, Glaring, Kersten, Koyama, et al., 2015). Nunamycin is a polar cyclic depsipeptide comprised of nine amino acid residues (aa) and containing a 3‐hydroxyl‐myristic acid side chain. Nunamycin shows potent activity against plant pathogens, in particular, the basidiomycete *R. solani* (Michelsen & Stougaard, 2011; Michelsen, Watrous, Glaring, Kersten, Koyama, et al., 2015). The second partially structurally elucidated peptide nunapeptin is composed of 22 aa residues attached to a fatty acid side chain and is active against ascomycetes notably *Fusarium* spp. and the oomycete *Pythium aphanidermatum* (Michelsen, Watrous, Glaring, Kersten, Koyama, et al., 2015). In addition to antifungal activity, both peptides have recently been shown to possess anticancer properties (Michelsen, Jensen, Venditto, Hennessy, & Stougaard, 2015). Nunamycin and nunapeptin biosynthesis is directed by nonribosomal peptide synthetase (NRPS) genes located on a large genomic island spanning over 100 kb. The genomic island shows some similarity to CLP biosynthetic regions found in other CLP‐producing pseudomonads and the genes can be grouped according to structural (*nunB1*,* nunB2*,* nunC*,* nunD*,* nunE*, n*upA*,* nupB*,* nupC*), regulatory (*nunF*,* nupR1*,* nupR2*), or secretory functions (*nunG*,* pseA*,* pseE*,* nupD*). In *Pseudomonas* spp., regulation of CLP synthesis can be mediated by the GacA/GacS system consisting of a sensor histidine kinase and a response regulator as shown for the regulation of amphisin synthesis in *Pseudomonas* sp. DSS73, or regulated by molecules such as *N*‐acyl‐homoserine lactones (*N*‐AHL) which mediate quorum sensing, for example, viscosin production in *P. fluorescens* PfA7B, or heat‐shock proteins such as DnaK or DnaJ, or alternatively CLP synthesis can be regulated by LuxR‐type transcriptional regulators (Dubern, Lagendijk, Lugtenberg, & Bloemberg, 2005; Nielsen, Nybroe, Koch, Hansen, & Soerensen, 2005; Dubern, Lugtenberg, & Bloemberg, 2006; Raaijmakers, de Bruijn, & De Kock, 2006; de Bruijn et al., 2009a; Song et al., 2015; Vaughn & Gross, 2016). CLPs produced by plant‐associated pseudomonads can be divided into six groups: viscosin, syringomycin, syringopeptin, amphisin, putisolvin, and tolaasin based on the similarity of the peptide sequence (Gross & Loper, 2009; Raaijmakers, de Bruijn, Nybroe, & Ongena, 2010; Roongsawang, Washio, & Morikawa, 2011). CLPs are a common feature of both plant beneficial and pathogenic pseudomonads playing a role in diverse bacterial functions (D'aes, De Maeyer, Pauwelyn, & Höfte, 2010; Flury et al., 2017). Nunamycin is similar in structure to syringomycin, a well‐characterized phytotoxin from *P. syringae*, as well as to thanamycin and cormycin from *Pseudomonas* sp. SHC52 and *P. corrugata*, respectively, whereas nunapeptin more closely resembles syringopeptin and corpeptin (Bender, Alarcon‐Chaidez, & Gross, 1999; Emanuele et al., 1998; Guenzi, Galli, Grgurina, Gross, & Grandi, 1998; Mo & Gross, 1991; Scaloni et al., 2004; Scholz‐Schroeder, Hutchison, Grgurina, & Gross, 2001; Zhang, Quigley, & Gross, 1997). Cormycin and corpeptin have been reported to be regulated by the *N*‐acyl‐homoserine‐lactone quorum sensing system, whereas there is limited knowledge of the genetic regulation of thanamycin and thanapeptin in *Pseudomonas* sp. SHC52 (Licciardello et al., 2012; van der Voort et al., 2015). In *P. syringae* pv. *syringae* B301D, production of the phytotoxins syringomycin and syringopeptin is controlled by the LuxR‐type regulatory genes *salA*,* syrF*, and *syrG* located on the *syr–syp* regulon (Lu, Wang, Wang, Chen, & Gross, 2005; Wang, Lu, Records, & Gross, 2006). LuxR‐like regulators are also involved in the production of viscosin by *P. fluorescens* SBW25 mediated by ViscAR and ViscBCR and similarly the massetolide biosynthesis genes of *P. fluorescens* SS101 have been shown to be regulated by a LuxR‐type transcriptional regulator (de Bruijn, de Kock, de Waard, van Beek, & Raaijmakers, 2008; de Bruijn et al., 2009a; Subramoni & Venturi, 2009; Song et al., 2015). In *P. fluorescens* In5, sequence analysis of the border regions flanking the *nun–nup* gene clusters also revealed genes encoding LuxR‐type transcriptional regulators. Two LuxR‐type regulator‐encoding genes designated *nupR1* and *nupR2* were identified downstream of the *nup* gene cluster and an additional LuxR‐type encoding gene *nunF* was found downstream of the *nun* gene cluster.

In order to begin unraveling regulation within the *nun–nup* gene clusters of *P. fluorescens* In5, the LuxR‐type regulator‐encoding gene *nunF* located downstream of the nunamycin biosynthesis genes was selected for characterization. Using a combination of insertional mutagenesis, gene expression and secondary metabolite profiling, the role of *nunF* in the production of the CLPs nunamycin and nunapeptin in *P. fluorescens* In5 was investigated. In addition, we investigated whether *nunF e*xpression is induced in response to either specific fungal‐associated carbon sources or root‐associated carbon sources present in the rhizosphere that can potentially indicate to the bacterium that it is in close vicinity to a fungal hyphae or a plant root. If the bacterium is specialized in using *nunF*‐regulated genes to induce hyphal leakages, it is hypothesized that the fungal‐associated carbon sources should result in a higher upregulation of *nunF*.

## 2 EXPERIMENTAL PROCEDURES

### 2.1 Strains, plasmids, and growth conditions

The strains and plasmids used in this work are listed in Table 1. All enzymes used in this study were from New England Biolabs (NEB) supplied by BioNordika, Herlev, Denmark. *Escherichia coli* DH5α^™^ (NEB, BioNordika) was used as the host strain for cloning procedures and broad‐host range plasmids were transferred to *P. fluorescens* strain In5 by electroporation as described previously by Michelsen, Watrous, Glaring, Kersten, Koyama, et al. (2015).

**Table 1:** Strains used in this study

Strains	Relevant characteristics	Reference
Plasmid		
pUX‐BF13	RK6 replicon – based helper plasmid; Ap^r^	Choi et al. 2006
pEX100T	*oriT*, *bla*, *sacB*	Michelsen et al. 2015
pEX100T::*nunF*	*oriT*, *bla*, *sacB, nunF* and arms for HR*, Ap^r^	This study
pEX100T::*nunF::Gm* ^r^	*oriT*, *bla*, *sacB, nunF,* GmR, Amp^r^	This study
pHN1270	broad ‐ host range plasmid, *ori*RK2, Apr^r^	Nakashima & Tamura, 2004
pHN1270::*nunF*	complementation of *nunF*	This study
pSEVA237R	broad ‐ host range plasmid, *ori*BBR1, Km^r^	Silva‐Rocha et al. 2013
pSEVA237R::*PnunF*	*nunF* promoter region fused to mCherry fluorescent protein, Km^r^	This study
Bacterial		
*Eschericia coli* DH5α^™^	*endA1 hsdR17 supE44 thi‐1 recA1 U169 deoR*	NEB, UK
*Pseudomonas fluorescens* In5	wild‐type	Michelsen et al. 2015
*P. fluorescens* In5	Δ*nunF*::Gm^r^	This study
*P. fluorescens* In5	5F5 *nunE*::Tn5Km^r^	Michelsen et al. 2015
*P. fluorescens* In5	M2D1 *nrps*::Tn5Km^r^	This study
*P. fluorescens* SS101	wild‐type	de Bruijn et al. 2008
Fungal and oomycete		
*Rhizoctonia solani* Ag3	wild‐type	Michelsen & Stougaard, 2011
*Pythium aphanidermatum*	wild‐type	Michelsen & Stougaard, 2011
*Fusarium graminearum PH1*	wild‐type	Frandsen et al. 2006
*Neurospora crassa 4200*	wild‐type	Colot et al. 2006

* HR; homologous recombination

### 2.2 Insertional mutagenesis of *nunF* by homologous recombination

Gene knockout by homologous recombination of *nunF* gene was carried out using the gene replacement vector, pEX100T (1). Primers used for insertional mutagenesis of *nunF* were primers 1: 5′‐GTATCGATTTGCGGGTTGGTC‐3′, 2: 5′‐CGGATTCTCTAGATTTCGTACGCTAC‐3′, 3: 5′‐GTAGCGTACGAAATCTAGAGAATCCG‐3′, and 4: 5′‐GGCTTTGCGGGTACTGCTG‐3′. Primers 2 and 3 introduced an *XbaI* site absent from the WT strain in *nunF* gene and the gentamicin resistance (Gm^r^) cassette was subsequently inserted at the newly introduced XbaI site. Fragments were amplified by polymerase chain reaction (PCR) using Phusion blunt‐end polymerase (annealing temperature, 63°C) according to the manufacturer's instructions (Fisher Scientific, Roskilde, Denmark). The blunt‐end‐amplified fragments were ligated into *Sma*I‐digested pEX100T as described previously by Michelsen, Watrous, Glaring, Kersten, Koyama, et al. (2015). The pEX100T vector with the *nunF* was disrupted by the Gm^r^ cassette amplified using PCR as above with forward and reverse primers and subsequently transformed into electrocompetent *P. fluorescens* In5 cells together with the pUX‐BF13 helper plasmid as described previously by Michelsen, Watrous, Glaring, Kersten, Koyama, et al. (2015).

### 2.3 Complementation of *nunF* knockout strain

Plasmid pHN1270 harboring the apramycin selectable marker was used as a complementation vector. The *nunF* gene was amplified from strain In5 genomic DNA by PCR using Phusion High Fidelity Polymerase (Fisher Scientific) and forward (5′‐GGAATTAACCATGCAGTGGTGGTGGTGGTGGTGCTCGAGAGGAGGACCGACCATGAATCG‐3′) and reverse (5′‐AATCTGTATCAGGCTGAAAATCTTCTCTCATCCGCCAAAACTAGTTTACGCCCCGATCATCCATTTG‐3′) primers to yield a 931‐bp fragment which was fused by Gibson Assembly^®^ (NEB) into pHN1270 linearized by *Nco*I and *Spe*I and transformed into *E. coli* DH5α^™^ (NEB). Fusion of the amplicon and plasmid was confirmed by restriction digest of plasmid DNA followed by Sanger sequencing (GATC‐Biotech, Konstanz, Germany) to confirm integrity of the DNA sequence. The resultant construct was then transformed into strain In5 by electroporation described earlier. Complementation was tested using the antifungal activity assay described below with the following strains: *P. fluorescens* In5 WT, *ΔnunF* with the control empty vector pHN1270 with or without IPTG (2 mmol/L) induction, and *ΔnunF* with the complementation plasmid pHN1270::*nunF* with or without IPTG (2 mmol/L) induction. Complementation was performed with biological triplicates and repeated twice.

### 2.4 Phenotypic analysis

Antifungal activity was assayed as described previously by Michelsen, Watrous, Glaring, Kersten, Koyama, et al. (2015). Briefly, a plug of *R. solani* Ag3 was placed in the center of small Petri dish (60Ø×15 mm) (Greiner Bio One, Frickenhausen, Germany) with a fifth potato dextrose agar (PDA; Difco, Lawrence, KS) and 10 μl of overnight culture of bacterial strains was spotted 2.5 cm away from the fungal plug. Where appropriate, 50 μg ml^−1^ and/or 10 μg ml^−1^ apramycin and gentamicin, respectively, were used and either 0 or 2 mmol/L IPTG (Fisher Scientific) was added. Plates were incubated at 20°C for 72 hr. Phenotypic analysis was conducted in triplicate (three biological replicates) and the experiment was repeated twice. Antifungal activity was determined as the percentage inhibition of radial growth (PIRG) as previously described by Michelsen, Watrous, Glaring, Kersten, Koyama, et al. (2015).

### 2.5 Growth, swarming, and biofilm formation assays

For monitoring growth, strain In5 and mutant strains were grown overnight in either LB or defined *Fusarium* medium (DFM) (Frandsen et al., 2006) supplemented with 0.5% wv^−1^ glucose, incubated with shaking at 200 rpm at 28°C. Cells were washed twice with 0.9% wv^−1^ NaCl and resuspended to an OD_600 nm_ = 0.1. A volume of 20 μl was added to a 96‐well microtiter plate containing 180 μl of LB or DFM 0.5% wv^−1^ glucose and growth was measured every hour for 30 hr in FLUOstar Omega Microplate Reader (BMG LABTECH, Offenburg, Germany). Swarming and biofilm formation assays were conducted as described by de Bruijn and Raaijmakers (2009a).

### 2.6 RNA isolation and first‐strand cDNA synthesis

Total RNA was extracted using the ZR Fungal/Bacterial RNA MiniPrep^™^ kit according to the manufacturer's instructions (Zymo Research, Nordic Biosite, Copenhagen, Denmark). Contaminating DNA was removed using the DNA‐free^™^ DNA removal kit (Fisher Scientific) according to the manufacturer's instructions. RNA purity and concentration were determined using Qubit^®^ RNA HR Assay Kit (Fisher Scientific) and RNA quality was assessed by agarose gel electrophoresis. First‐strand cDNA synthesis was performed using ProtoScript^®^ first‐strand synthesis kit (NEB) according to the manufacturer's instructions.

### 2.7 Quantification of expression of genes from *nun* and *nup* gene clusters

Expression of the housekeeping gene *gyrB* and target genes *nunB1*,* nunB2*,* nunD*,* nunE*,* nupA*,* nupB*, and *nupC* were quantified by qRT‐PCR using the Stratagene Brilliant III SYBR Green QPCR Master Mix (Agilent Technologies, AH Diagnostics, Aarhus, Denmark) according to the manufacturer's instructions. The primers used for amplification are listed in Table 2. A standard curve was prepared as a 10‐fold serial dilution for each primer set to verify efficiency prior to conducting qRT‐PCR analysis. RT products were PCR amplified in a 25‐μl volume reaction containing 12.5 μl Brilliant III SYBR Green QPCR Master Mix and 200 nmol/L of each of forward and reverse transcript‐specific primers (Table 2). PCR reactions were conducted in a Stratagene Mx3000^™^ real‐time PCR machine (Stratagene, La Jolla, CA) and the program consisted of 1 cycle 95°C 10 min, 40 cycles 95°C 30 s, 58°C 1 min, 72°C 30 s followed by 1 cycle 95°C 1 min, 55°C 30 s, and 95°C 30 s according to the manufacturer's instructions (Agilent Technologies). Gene expression was calculated as the threshold cycle (*C*_T_) values obtained from qRT‐PCR which were then used to calculate the accumulation of the target gene (relative mRNA accumulation) relative to the housekeeping *gyrB* transcript by the 2^−ΔΔCT^ method, where ΔΔ*C*_T_ = (*C*_T_, target gene‐*C*_T_, housekeeping gene) (Livak & Schmittgen, 2001). qRT‐PCR was performed in duplicate (technical replicates) on three independent RNA isolations (biological replicates).

**Table 2:** Primers used in this study for qRT‐PCR

Gene^a^	Primer sequence (5′–3′)	Product size (bp)
*gyrB*	For‐CAGAGGTCGAACTGATCTC	170
	Rev‐CTCGGTCACTGGCTTCTTG	
*nunB1*	For‐GAGTGCCTGGACCGAACAATC	194
	Rev‐GCTCGGTAAGCCCTGATTGC	
*nunB2*	For‐GAGCTATGACCCCGAGAACG	162
	Rev‐CAGAAAATGATGAACTGGCC	
*nunD*	For‐GTTCAGTAGCCTGTTCAACTATC	140
	Rev‐GGTCATGTTGGTAATCGTTGACC	
*nunE*	For‐GTTCCAGGTGATGTTAAGCC	183
	Rev‐GCGATATCGAACAGATCAC	
*nupA*	For‐GCATTGCCGGGGAAATCAAC	200
	Rev‐CTTCACCTGGAAGTCGTTAC	
*nupB*	For‐CACATTACGCCTCCATCTGC	199
	Rev‐CCACAGCACCGAGAGTTTCG	
*nupC*	For‐CGATGCCACAGCCTTCAGTC	167
	Rev‐GATGTTCAGGTTGATAGACCG	
*nupR1*	For‐GAGCATGAATTCTTGCACTG	158
	Rev‐GTAGAGCGCTTTGATCATGG	
*nupR2*	For‐CGCCACAGGAATGTGCCTTC	199
	Rev‐GAGAGGGTCAATCCCGATTC	
*nupP*	For‐CAAGCAATGGTTCTTCTGCG	154
	Rev‐GACGCCCTTAGTGAAGGTG	

a Gene, nucleotide sequence data are available from the *Pseudomonas fluorescens* In5 antifungal genomic region deposited under GenBank accession number KCC880158 and the accession number for the housekeeping gene *gyrB* is HM070426.

### 2.8 Peptide extraction

Strains were grown as described previously for phenotypic analysis. The agar and biomass from a single plate were transferred to a Falcon tube and 10 mL 2‐butanol containing 1% formic acid was added. The tubes were shaken and then placed in an ultrasonic bath for 30 min before being subjected to centrifugation at 4,000*g* for 10 min. The organic phase was removed and then dried under a constant stream of nitrogen. The residue was resuspended in 300 μl of methanol, subjected to centrifugation at 4,000*g* for 10 min, and then 250 μl of the solution was collected for LC‐HRMS analysis.

### 2.9 Liquid chromatography–high‐resolution mass spectrometry analysis

Ultrahigh‐performance liquid chromatography–diode array detection–quadruple time of flight mass spectrometry (UHPLC‐DAD‐QTOFMS) was performed on an Agilent Infinity 1290 UHPLC system (Agilent Technologies, Santa Clara, CA) equipped with a diode array detector. Separation was obtained on an Agilent Poroshell 120 phenyl‐hexyl column (2.1 × 250 mm, 2.7 μm) with a linear gradient consisting of water (A) and acetonitrile (B) both buffered with 20 mmol/L formic acid, starting at 10% B and increased to 100% in 15 min where it was held for 2 min, returned to 10% in 0.1 min, and keeping it for 3 min (0.35 mL/min, 60°C). An injection volume of 1 μl was used. MS detection was performed on an Agilent 6545 QTOF MS equipped with Agilent Dual Jet Stream electrospray ion source with the drying gas temperature of 250°C and gas flow of 8 L/min and sheath gas temperature of 300°C and flow of 12 L/min. Capillary voltage was set to 4,000 V and nozzle voltage to 500 V. Mass spectra were recorded as centroid data for *m*/*z* 85–1,700 in MS mode and *m*/*z* 30–1,700 in MS/MS mode, with an acquisition rate of 10 spectra/s. Lock mass solution in 70:30 methanol:water was infused in the second sprayer using an extra LC pump at a flow of 10–50 μl/min, the solution contained 1 μmol/L tributylamine (Sigma‐Aldrich), 10 μM Hexakis(2,2,3,3‐tetrafluoropropoxy) phosphazene (Apollo Scientific Ltd., Cheshire, UK) as lock masses. The [M + H]+ ions (*m*/*z* 186.2216 and 922.0098, respectively) of both compounds was used.

Analysis was performed using Agilent MassHunter version B.07.00. Data files were examined for the presence of nunamycin (C_47_H_80_ClN_11_O_19_) and nunapeptin (C_95_H_157_N_23_O_26_ and C_94_H_155_N_23_O_26_). LC‐HRMS analysis was performed in triplicate (technical replicates) on three experiments representing three independent peptide extractions (biological replicates).

### 2.10 Construction of a mCherry‐based reporter of *nunF* gene expression

The reporter strain *P. fluorescens* In5 harboring the *nunF* gene promoter region cloned in front of mCherry was previously constructed (Hennessy, Stougaard, & Olsson, 2017). Briefly, a fragment containing the predicted promoter region upstream of the *nunF* start codon was amplified using LongAmp^™^ *Taq* DNA polymerase (NEB) from strain In5 genomic DNA; the PCR product was then cloned using Gibson Assembly^®^ (NEB) into pSEVA237R (*RK2*‐Km^R^‐mCherry) upstream of an mCherry‐expressing cargo. The resultant construct was then transformed into strain In5 by electroporation as described earlier.

### 2.11 Analysis of *nunF* gene expression in vitro

For monitoring *nunF* gene expression in vitro, strain In5 carrying *nunF*–*mCherry* on pSEVA237R was grown overnight in a minimal media (DFM) supplemented with 0.5% wv^−1^ glucose and 25 μg ml^−1^ kanamycin with shaking 200 rpm at 28°C. Cells were washed twice with 0.9% wv^−1^ NaCl and resuspended to an OD_600 nm_ = 0.1 and 20 μl was added to a 96‐well microtiter plate, together with 180 μl of DFM supplemented with 0.05% wv^−1^ of different carbon sources. The following carbon sources were used: (1) “fungal‐specific” carbon sources that can be expected in the vicinity of hyphae or with compromised leaky membranes: laminarin and laminartriose (outer cell wall) (Brown & Gordon, 2003; Fesel & Zuccaro, 2016; Klarzynski et al., 2000; Trouvelot et al., 2014), trehalose (sugar accumulating with membrane stress) (Hallsworth, 1997; Bhaganna et al., 2010; Wyatt et al., 2015; glycerol (accumulating in response to stress from loss of turgor), citrate and oxalate (organic acids often secreted in large amounts by hyphae) (Bravo et al., 2013; Deveau et al., 2010; Gadd, 2007; Scheublin, Sanders, Keel, & van der Meer, 2010). (2) “Root‐specific” carbon sources expected to be present in the rhizosphere: cellobiose (as degradation product of cellulose) (Kumar, Kuzyakov, & Pausch, 2016), arabinose, raffinose, fucose (root exudates)(Gunina & Kuzyakov, 2015). (3) Other carbon sources like inositol and especially glucose were used as controls since most organisms are stimulated by these. Growth and fluorescence of mCherry was measured every hour for 48 hr in FLUOstar Omega Microplate Reader (BMG LABTECH). Analysis of *nunF* gene expression was performed using biological triplicates. Increase in mCherry responses faster than glucose was seen as a preferential *nunF* regulation in response to that carbon source.

### Data analysis

Statistical analysis of data was conducted using one‐way ANOVA or paired *t*‐test for normally distributed data or the Kruskal–Wallis *H* test and Mann–Whitney test for nonparametrical data using Minitab (Minitab release © 16, 2011 Minitab Inc.). If possible, non‐normally distributed data was transformed to fit a normal distribution using Johnson transformation within Minitab.

## 3 RESULTS

### 3.1 Genomic context of *nunF* in *P. fluorescens* In5

**Figure 1:**
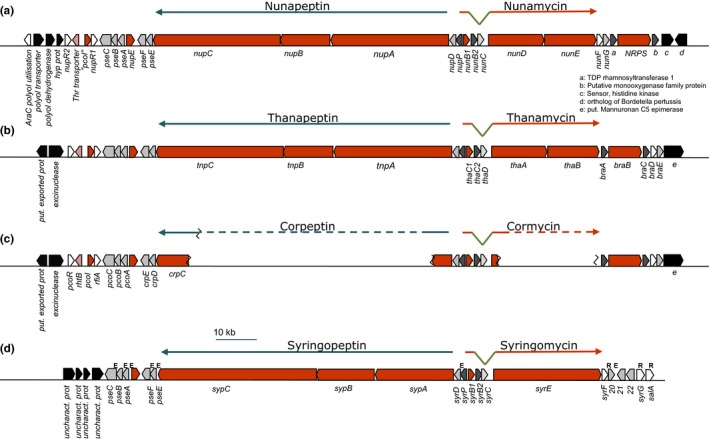
Organization of the *nun* and *nup* gene clusters in *Pseudomonas fluorescens* In5 and comparison with other pseudomonads. Map of the nunamycin (*nun*) and nunapeptin (*nup*) gene clusters located on a genomic island spanning over 100 kb in strain In5. Gene clusters harbor *nunF* and all the known biosynthetic genes for nunamycin and nunapeptin, including the left and right border regions (a) and comparison with the thanapeptin and thanamycin gene clusters from *Pseudomonas* sp. SHC52 (b) and the corpeptin and cormycin gene clusters from *P. corrugata* CFBP5454 (c) and the syringopeptin and syringomycin gene clusters from *P. syringae* pv. *syringae* B738a (d). The solid orange arrows indicate the biosynthesis genes, and regulatory genes are depicted by white arrows, whereas genes with secretory functions are represented by gray arrows with additional genes shown in dark gray arrows

**Figure 2:**
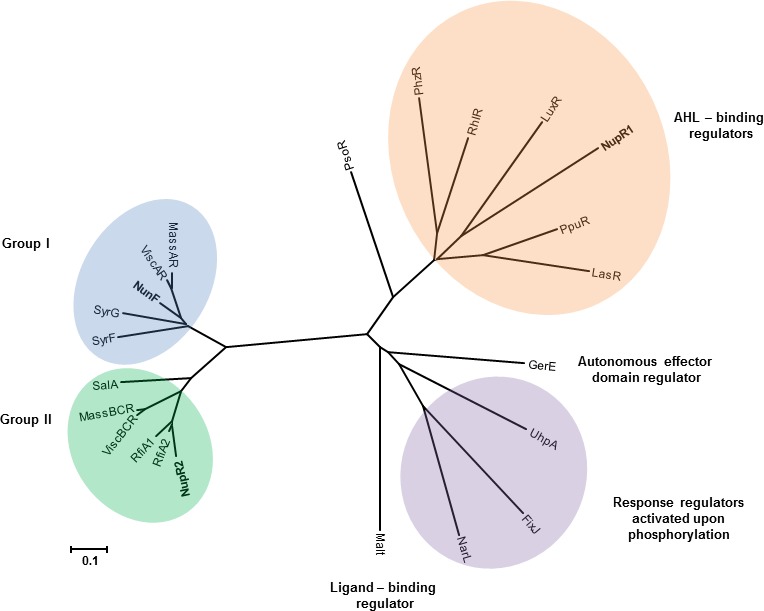
Phylogenetic analysis of LuxR‐type regulators flanking the *nup*–*nun* gene clusters in *Pseudomonas fluorescens* In5. A phylogenetic tree was constructed using MEGA 6.0 (Tamura, Stecher, Peterson, Filipski, & Kumar, 2013) based on the amino acid sequence of LuxR‐type regulators from strain In5 and other well‐characterized LuxR‐type regulators. The tree has been assigned different groups (adapted from de Bruijn et al., 2009a) based on primary structure (Groups I and II) and functions of neighboring genes. Abbreviations for organisms and their regulators are listed alphabetically as follows: FixJ, *Sinorhizobium meliloti* (NP_435915); GerE, *Bacillus subtilis* (NP_390719); LasR, *Pseudomonas aeruginosa* (BAA06489); LuxR, *Vibrio fischeri* (AAQ90196), NunF, *P. fluorescens In5* (WP_054049653); NupR1, *P. fluorescens* In5 (WP_054050468); NupR2, *P. fluorescens* In5 (WP_054050462); MalT, *Escherichia coli* (AAA83888); MassAR,* P. fluorescens* SS01 (ABW87979); MassBCR,* P. fluorescens* SS01 (ABW87989); NarL, *E. coli* (CAA33023); PhzR, *P. chlororaphis* (ABR21211); PpuR, *P. putida* (AAZ80478); PsoR, *P. fluorescens* A506 (WP_014717607); RhIR,* P. aeruginosa* (NP_252167); RfiA1, *P. brassicearum* (WP_025213903); RfiA2, *P. corrugata* (WP_024779115); ViscAR,* P. fluorescens* SBW25 (WP_015884801); ViscBCR,* P. fluorescens* SBW25 (WP_043205227); SalA, *P. syringae* pv. *syringae* (WP_016568164); SyrF, *P. syringae* pv. *syringae* (WP_016568170); SyrG, *P. syringae* pv. *syringae* (WP_016568165); UhpA, *Salmonella enterica* serovar Typhimurium (NP_462689)

In previous studies, whole genome sequencing and genome walking of transposon insertion sites led to the identification of a large NRPS‐encoding genomic island for CLP biosynthesis from *P. fluorescens* In5 (Michelsen, Watrous, Glaring, Kersten, Koyama, et al., 2015). The genomic island which spans approximately 125 kb consists of two gene clusters: one for nunamycin biosynthesis and another for nunapeptin biosynthesis. The nunamycin (*nun*) gene cluster consists of eight open‐reading frames (ORFs) spanning approximately 38 kb, whereas the nunapeptin (*nup*) gene cluster spans approximately a 87‐kb region of 14 ORFs (Figure 1). Analysis of the genes adjacent to the NRPS genes directing either nunamycin (*nunD*,* nunE*) or nunapeptin (*nupA*,* nupB*,* nupC*) identified three LuxR regulatory‐like genes flanking either the *nun* (*nunF*) or *nup* (*nupR1* and *nupR2*) regulon. Subsequent alignment and Pfam analysis of the putative regulatory genes showed that the C‐terminal regions of *nunF*,* nupR1*, and *nupR2* are relatively conserved and contain the DNA binding HTH signature motif of LuxR‐type transcriptional regulators (Finney, Blick, Murakami, Ishihama, & Stevens, 2002; Figure S1). However, only NupR1 contained the *N*‐AHL‐binding domain (2–118 residues) found in the *Vibrio fischeri* LuxR gene (Shadel & Baldwin, 1991). None of the three genes contained the FixJ regulator domain which is required for protein activation upon phosphorylation. Phylogenetic analysis of the three putative LuxR‐type regulators from the *nun*–*nup* gene clusters showed that the regulators did not cluster according to location (upstream or downstream of biosynthesis genes) as previously observed for LuxR‐type regulators flanking the massetolide or viscosin biosynthesis genes (de Bruijn & Raaijmakers, 2009a) (Figure**** 2). Instead, the LuxR‐type regulators (NunF, NupR1, and NupR2) were dispersed among the different groups (Figure**** 2). All LuxR‐type regulators analyzed from In5 were clustered distantly from well‐characterized LuxR‐type regulators (e.g., FixJ, RhIR, LasR, and LuxR) with the exception of NupR1 suggesting that they could be classified as a separate group along with the massetolide LuxR‐type regulators (MassAR, MassBCR) and viscosin LuxR‐type regulators (ViscAR, ViscBCR) as previously proposed by de Bruijn et al. (2009a) (Figure**** 2). NunF clustered into assigned Group I together with SyrF which has been characterized and shown to be a key regulator of syringomycin production and also playing a role in syringopeptin synthesis in *P. syringae* pv. *syringae* and is located adjacent to the syringomycin biosynthesis genes similarly to NunF. While the genomic island gene organization is very similar to that of other pseudomonads, a notable difference compared to *P. syringae* is the absence of the gene encoding SalA. In *P. syringae*, SalA together with SyrF and SyrD form part of a complex regulatory network involved in the biosynthesis and secretion of both syringomycin and syringopeptin in addition to syringolin (Wang et al., 2006) (Figure 1).

In strain In5, the *nunF* gene is 831 bp with 57–82% identity to homologs in other *Pseudomonas* genomes. The *nunF* gene encodes a LuxR‐type transcriptional regulator showing the strongest homology (65% percentage identity [PID] at the protein level) to LuxR family transcriptional regulators from *P. batumici*,* Pseudomonas* sp. SHC52, and *P. corrugata*. Of the characterized LuxR‐type regulators shown to be involved in CLP regulation, NunF showed the strongest homology to SyrF from *P. syringae* pv. *syringae* B301D (Table S1). As mentioned previously, the NunF protein (277 aa) consists of the HTH DNA binding domain (217–273 residues). Similarly to the *syr* and *syp* gene clusters of *P. syringae* pv. *syringae* B301D, three genes encoding LuxR‐type regulators were previously identified flanking the peptide biosynthetic genes: *syrF*,* salA*, and *syrG* (Lu, Scholz‐Schroeder, & Gross, 2002). NunF shows 63% PID to SyrF and 53% PID to SyrG. The SalA DNA binding transcriptional regulator showed 38% PID to NunF and 46% PID to NupR2 in strain In5. Consistent with previous studies, phylogenetic analysis of the three LuxR‐type regulators located upstream (*nupR1*,* nupR2*) of the *nun* or *nup* gene clusters clustered differently from NunF located downstream of the *nun* regulon (Figure 2). To elucidate if *nunF* encoding a LuxR‐type protein plays a role in antimicrobial activity and production of nunamycin and nunapeptin, targeted knockout of *nunF* and subsequent mutant characterization was conducted.

### 3.2 Role of *nunF* in growth, antimicrobial activity, biofilm formation, and swarming motility of *P. fluorescens* In5

**Figure 3:**
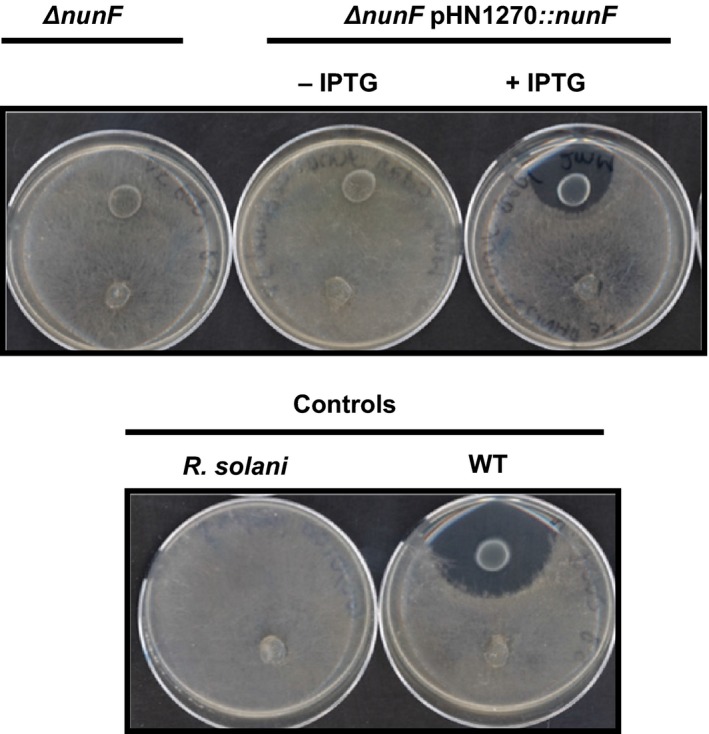
Antifungal activities of *nunF* knockout mutant and complementation strains. *Pseudomonas fluorescens* strain In5 wild‐type (WT), *nunF* knockout mutant (*ΔnunF*), *nunF* knockout mutant harboring empty vector (*ΔnunF* pHN1270), or *nunF* knockout mutant expressing *nunF* (*ΔnunF* pHN1270::*nunF*) were co‐cultured with the basidiomycete *Rhizoctonia solani* strain Ag3. Bacteria (10 μl) were spotted 2.5 cm away from a fungal plug placed on potato dextrose agar (PDA) fifth strength and incubated 72 hr at 20°C

To confirm the role of *nunF* in antimicrobial activity, insertional mutagenesis to replace *nunF* by an antibiotic selectable marker was performed. Phenotypic analysis of the *nunF* mutant compared to the wild‐type strain (WT) and two reference mutant phenotypes with reduced antimicrobial activity and defective in either nunapeptin (M2D1) or nunamycin (5F5) biosynthesis showed a loss of antimicrobial activity against all three pathogens tested: *R. solani*,* P. aphanidermatum*, and *F. graminearum* in addition to the model system fungus *N. crassa*, indicating the potential role of *nunF* in both nunamycin and nunapeptin synthesis (Table 3). The *nunF* mutation did not affect growth of strain In5 as the *nunF* knockout mutant showed comparable growth to that of the WT as did mutants M2D1 and 5F5 (Figure S2). The role of CLPs in biofilm formation has been widely reported in pseudomonads (Bonnichsen et al., 2015; de Bruijn & Raaijmakers, 2009a). To investigate whether a *nunF* mutant and mutants defective in peptide production (M2D1 and 5F5) played a role in biofilm formation, the crystal violet method in microtiter plates was used with *P. fluorescens* SS101 as a reference strain (de Bruijn & Raaijmakers, 2009b) (Figure S3). Compared to WT, all three mutants showed a significant reduction in biofilm formation with the *nunF* mutant the lowest biofilm‐forming strain at 4 hr (*p *<* *.001) (Figure S3A). There was no significant difference between planktonic cells among strains (*p *>* *.05) (Figure S3B). Swarming motility was also tested (Table S2). Compared to the reference strain *P. fluorescens* SS101, In5 showed a featureless swarming phenotype and not dendritic. Swarming was observed only on 0.25% agar where the *nunF* mutant showed a significant reduction in swarming ability compared to WT and mutant strains (*p *<* *.05). Previous observations have shown that nunamycin and nunapeptin are key components of the antimicrobial activity of strain In5. To confirm the role of *nunF* in antimicrobial activity, complementation of the *nunF* mutant of strain In5 was performed using the low‐copy, broad‐host range vector pHN1270::*nunF* which restored antifungal activity when tested against *R. solani*, whereas the empty vector control pHN1270 had no effect (Figure**** 3).

**Table 3:** Antifungal and anti‐*Pythium* activity of *Pseudomonas fluorescens* In5 and mutants defective in CLP production

Fungus/Bacteria	WT	5F5	M2D1	*ΔnunF*
*R. solani*	73.19 ± SE 0.47	0.0 ± SE 0.0	72.97 ± SE 0.17	0.0 ± SE 0.0
*F. graminearum*	56.21 ± SE 0.43	54.75 ± SE 0.80	0.0 ± SE 0.0	0.0 ± SE 0.0
*P. aphanidermatum*	54.13 ± SE 0.64	56.55 ± SE 2.39	0.0 ± SE 0.0	0.0 ± SE 0.0
*N. crassa*	74.73 ± SE 1.74			

*Pseudomonas fluorescens* strain In5 wild‐type (WT), *nunF* knockout mutant (*ΔnunF*), mutant M2D1, and mutant 5F5 were co‐cultured with the basidiomycete *Rhizoctonia solani* strain Ag3, the ascomycetes Fusarium graminearum PH‐1 and Neurospora crassa 4200, and the oomycete *Pythium aphanidermatum*. Bacteria (10 μl) were spotted 2.5 cm away from a fungal plug placed on potato dextrose agar (PDA) fifth strength and incubated 72 hr at 20°. Percentage inhibition of growth (PIRG) was calculated as described by Michelsen, Watrous, Glaring, Kersten, Koyama, et al. (2015). Standard error (SE) was calculated across biological triplicates.

### 3.3 Transcriptional analysis of the *nunF* mutant of *P. fluorescens* In5

**Figure 4:**
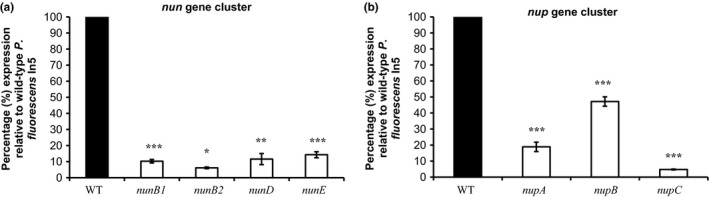
Transcriptional analysis of *Pseudomonas fluorescens* In5 *nunF* mutant. qRT‐PCR was conducted on RNA samples harvested at 24 hr from strains incubated on fifth strength PDA at 20°C. mRNA accumulation of genes catalyzing NRPs synthesis (*nunB1*,* nunB2*,* nunD*,* nunE*,* nupA*,* nupB*, and *nupC*) was quantified relative to the housekeeping gene encoding DNA gyrase subunit B (*gyrB*) (HM070426) and the percentage mRNA accumulation of the *nunF* mutant relative to the WT representing 100% was calculated. Quantitative RT‐PCR (qRT‐PCR) was conducted twice with biological triplicates. Error bars represent the standard error of the means. mRNA accumulation significantly different from WT strain is highlighted with an asterisk (level of significance: *<.05, **<.01, ***<.001)

**Figure 5:**
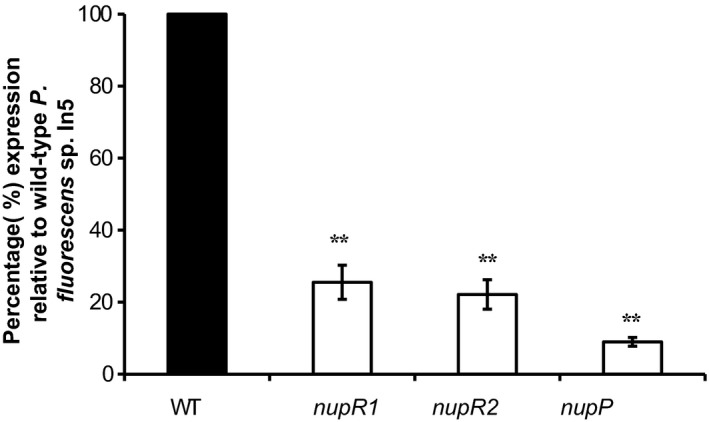
Effect of a *nunF* single‐gene deletion on transcription of the *nupP*,* nupR1*, and *nupR2* genes putatively encoding transcriptional regulators located adjacent to the nunapeptin biosynthesis genes. qRT‐PCR was conducted on RNA samples harvested at 24 hr from strains incubated on fifth strength PDA at 20°C. mRNA accumulation of genes putatively encoding the transcriptional regulators *nupR1* and *nupR2* in addition to *nupP* located upstream of the nunapeptin biosynthetic genes was quantified relative to the housekeeping gene encoding DNA gyrase subunit B (*gyrB*) (HM070426) and the percentage mRNA accumulation of the *nunF* mutant relative to the WT representing 100% was calculated. Quantitative RT‐PCR (qRT‐PCR) was conducted twice with biological triplicates. Error bars represent the standard error of the means. *Pseudomonas fluorescens* In5 *nunF* single‐gene deletion strain (*ΔnunF*) *nupP*,* nupR1*, and *nupR2* mRNA accumulation significantly different from WT strain is highlighted with an asterisk (level of significance: *≤.05, **≤.01, ***≤.001)

NRPs are synthesized by nonribosomal peptide synthetases (NRPS) which form large megaenzyme assembly lines for peptide production. Quantitative RT‐PCR analysis was used to analyze transcript levels of CLP biosynthesis genes in WT In5 compared to the *nunF* mutant under CLP‐inducing conditions. As expected, significantly reduced transcript levels were recorded for all genes catalyzing both nunamycin (*nunD*,* nunE*) and nunapeptin (*nupA*,* nupB*,* nupC*) biosynthesis in the *nunF* mutant compared to WT (*p *<* *.001) (Figure**** 4). Two additional transcripts *nunB1* and *nunB2* also involved in catalyzing nunamycin synthesis were significantly downregulated in the *nunF* mutant (*p *<* *.05) (Figure 4a). The *nunB1* transcript which is thought to be involved in selection and activation of Thr‐9 based on similarity to *thaC1* and *syrB1* in *Pseudomonas* sp. SHC52 and *P. syringae* pv. *syringae*, respectively, was downregulated 10‐fold in the *nunF* mutant compared to the WT. In addition, the *nunB2* transcript with homology to *thaC2* and *syrB2* was downregulated 16‐fold in the *nunF* mutant compared to WT (Figure 4a). Similarly to nunamycin, the nunapeptin biosynthesis genes *nupA*,* nupB*,* nupC* were significantly downregulated in the *nunF* mutant compared to WT (Figure 4b). Interestingly, the expression of *nupA* and *nupB* compared to the WT were 19% and 47%, respectively, suggesting that a more complex regulation is involved in nunapeptin synthesis that may not solely rely on *nunF*. In order to investigate the effect of a *nunF* knockout on transcription of additional genes putatively encoding regulators located adjacent to NRPs biosynthesis genes, qRT‐PCR analysis was also used to analyze the transcript levels of *nupP*,* nupR1*, and *nupR2* in strain In5 WT compared to the *nunF* single‐gene knockout mutant (Figure**** 5). A significant reduction in the expression of *nupP*,* nupR1*, and *nupR2* was observed in the *nunF* mutant strain compared to the WT suggesting that *nunF* interacts with the two putative regulators located on the NRPS genomic island downstream of the nunapeptin biosynthetic genes and possibly also *nupP* located upstream of the *nup* genes.

### 3.4 NunF is required for CLP biosynthesis in *P. fluorescens* In5

**Figure 6:**
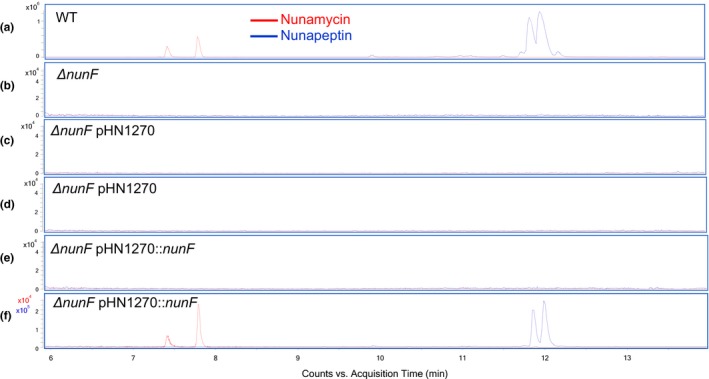
Liquid chromatography–high‐resolution mass spectrometry (LC‐HRMS) analysis of *Pseudomonas fluorescens* In5 WT,* ΔnunF*, and complemented mutant. Strains of *P. fluorescens* In5 WT (a), *ΔnunF* (b), *ΔnunF* pHN1270 0 mmol/L IPTG (c), *ΔnunF* pHN1270 2 mmol/L IPTG (d), *ΔnunF* pHN1270::*nunF* 0 mmol/L IPTG (e), and *ΔnunF* pHN1270::*nunF* 2 mmol/L IPTG (f) were grown on fifth strength PDA for 48 hr at 20°C. Peptide extraction was performed on agar and biomass using 2‐butanol containing 1% formic acid followed by ultrasonication and evaporation of the organic phase under nitrogen. Samples were resuspended in methanol. LC‐HRMS analysis was performed in triplicate. Extracted ion Chromatograms (EICs) of proton adducts of nunamycin (red) and nunapeptin (blue)

LC‐HRMS confirmed that the *nunF* mutant did not produce nunamycin or nunapeptin (Figure 6) in detectable quantities. Analysis of extracts from strains grown on fifth strength PDA required to induce CLP production, detected both nunamycin and nunapeptin in the WT and complemented *nunF* knockout extracts (*ΔnunF* pHN1270::*nunF*), and neither peptide was produced in the *nunF* knockout empty vector (*ΔnunF* pHN1270) control (Figure**** 6). Based on these results, *nunF* is required for both nunamycin and nunapeptin biosynthesis in strain In5. As *nunF* is putatively encoding a LuxR‐type regulator, the presence of *N*‐acyl‐homoserine lactones (AHLs) was also investigated. The presence of *N*‐hexanoyl‐homoserine lactone was confirmed by accurate mass (observed *m/z*: 200.1279 for [M + H]^+^ C_10_H_17_NO_3_^+^, mass deviation: 1.1 ppm) as well as retention time and tandem MS spectrum, particularly the diagnostic fragment at *m/z* 102.0549 (Kildgaard et al., 2014). This AHL was observed in all extracts, regardless of the growth medium or the presence/absence of the cyclic peptides.

### 3.5 Dynamics of *nunF* gene expression in *P. fluorescens* In5

**Figure 7:**
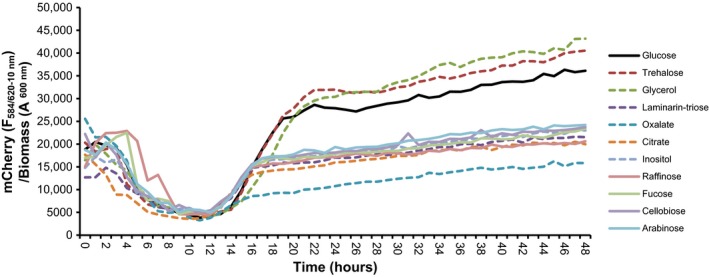
Analysis of the temporal response of the *nunF* promoter to rhizosphere‐associated carbon sources using a *nunF* promoter mCherry reporter strain. A *nunF* promoter fusion to mCherry was constructed and used as a reporter system in *P. fluorescens* In5 for monitoring *nunF* gene expression in vitro. *Pseudomonas fluorescens* In5 reporter strain (pSEVA237R::*PnunF*::*mCherry*) and the control strain (pSEVA237R::*mCherry*) were screened in a 96‐well microtiter plates with varying rhizosphere‐associated carbon sources. Dashed lines represent fungal‐associated carbon sources and nondashed show plant‐associated carbon sources with glucose control in black. Growth and fluorescence of mCherry was measured every hour for 48 hr in FLUOstar Omega Microplate Reader (BMG LABTECH). Graph shows mCherry signal from the reporter strain (pSEVA237R::*mCherry*) over the control strain (empty vector; (pSEVA237R::*mCherry*)

In order to investigate whether environmental factors play a role in regulating *nunF* expression, a reporter strain harboring the *nunF* promoter fused to mCherry was assayed in microtiter plates for growth and mCherry expression recorded on carbon sources indicating the presence of a plant rhizosphere or indicating the presence of a fungus and compared to glucose controls (Figure 7 and S4). The mCherry expression with the “fungal carbon sources” glycerol and trehalose was shown to be higher than with “plant carbon sources” like arabinose and cellobiose (Figure 7, Table S3 and Figure S4).

## 4 DISCUSSION

The genomic island of *P. fluorescens* In5 harboring two large gene clusters required for synthesis of two CLPs nunamycin and nunapeptin, encodes three genes encoding putative LuxR‐type transcriptional regulators, of which *nunF* was characterized in this study. The present findings demonstrate that the LuxR‐like protein NunF is involved in the antimicrobial activity and regulation of the two CLPs nunamycin and nunapeptin in strain In5.

In *Pseudomonas* spp., the closest characterized homolog of NunF is the SyrF protein from *P. syringae* pv. *syringae* strain B301D and the recently characterized SyrF protein from the causal agent of brown spot disease on bean *P. syringae* pv. *syringae* B728a (Vaughn & Gross, 2016). All three proteins have no defined N‐terminal regulatory domain, but in contrast have a highly conserved C‐terminal domain marked by a HTH DNA binding domain (Aravind, Anantharaman, Balaji, Babu, & Iyer, 2005; Vaughn & Gross, 2016). Phylogenetic analysis showed that NunF together with SyrF in addition to SalA and SyrG from *P. syringae* pv*. syringae* strains could be classified into a new subfamily of LuxR proteins as previously proposed (de Bruijn & Raaijmakers, 2009a; Jacobs et al., 2003). This family would also include the viscosin and massetolide LuxR regulators described for *P. fluorescens* strains SS101 and SBW25 (de Bruijn & Raaijmakers, 2009a).

The importance of NunF with regard to antimicrobial activity against three model pathogens representing different major groups (ascomycete, basidiomycete, and oomycete) was demonstrated using dual‐culture interaction assays. In a previous study, nunamycin and nunapeptin were shown to be key components of the antimicrobial activity of In5 (Michelsen, Watrous, Glaring, Kersten, Koyama, et al., 2015). This observation was further supported by the reduction in pathogenicity toward fungi and the oomycete *Pythium* by mutant M2D1, defective in nunapeptin production and mutant 5F5 defective in nunamycin synthesis. Furthermore, M2D1 showed no significant reduction in antagonism toward *R. solani* compared the WT and in contrast the anti‐*Fusarium* and anti‐*Pythium* activity of mutant 5F5 was comparable with the WT. This observation supports previous work proposing nunapeptin to be potent against *Fusarium* spp., and *Pythium* in contrast to nunamycin which is more effective in the growth inhibition of *R. solani* (Michelsen, Watrous, Glaring, Kersten, Koyama, et al., 2015). Interestingly, interactions between the In5 strains and *N. crassa* produced phenotypes similar to those observed for *R. solani* and not for *F. graminearum* as would have been expected as both fungi belong to the Ascomycota phylum. It is important to note that the M2D1 and 5F5 were generated by random transposon insertion which may have polar effects on the expression of neighboring genes (Jacobs et al., 2003). The *nunF* mutant generated by insertional‐directed mutagenesis when screened in a dual‐culture assay showed a complete loss of antimicrobial activity against all pathogens tested. This was expected based on the phenotypic analysis of M2D1 and 5F5, but surprising when compared to previous studies in *P. syringae* pv. *syringae* where a mutant in *syrF* showed a 61% reduction in virulence in *P. syringae* pv. *syringae* B728a and 83% in *P. syringae* pv. *syringae* B310D, respectively (Lu et al., 2002; Vaughn & Gross, 2016). This loss of antagonism toward fungi and *Pythium* can be attributed to the loss in production of both nunamycin and nunapeptin as validated by LC‐HRMS analysis. The *nunF* mutant did not produce detectable levels of nunamycin or its derivatives and also did not produce nunapeptin or its derivatives. In contrast to strain In5, where a functional copy of *nunF* is required for production of both peptides, *syrF* appears to be critical for syringomycin production in *P. syringae* pv. *syringae* B301D and to a lesser extent syringopeptin production (Lu et al., 2002). Nunamycin and nunapeptin production were restored when a functional copy of *nunF* was expressed on a broad‐host range RK2 *ori* complementation plasmid. Levels were not comparable to the WT correlating with the dual‐culture assay where the complemented *nunF* mutant showed a restoration of antifungal activity of 79%. This could be the result of using a synthetic RBS and spacer region and low copy complementation vector. Partial restoration of nunamycin and nunapeptin is in accordance with complementation studies described in *P. syringae* pv. *syringae* B728a where partial restoration of syringomycin was reported following expression of *syrF* in *trans* (Vaughn & Gross, 2016).

Quantitative real‐time PCR was used to analyze the effect of a *nunF* deletion on both nunamycin and nunapeptin biosynthesis genes. In the *nunF* mutant, all the structural genes required for either nunamycin or nunapeptin synthesis were negatively affected indicating that the *nunF* is part of the *nun*–*nup* regulon in strain In5. Although in *P. syringae* pv. *syringae* B301, SalA regulates syringomycin and syringopeptin through the activation of *syrF*, in strain In5, it would appear that NunF directly activates transcription of the NRPS genes driving nunamycin and nunapeptin synthesis. Gene expression analysis showed that a mutation in *nunF* did significantly affect transcription of the two additional regulator‐like genes located on the CLP genomic island suggesting that *nunF* may also control expression of NupR1/R2. Further studies would be required to determine whether one or both of these putative regulators directly affect cluster expression and whether the loss of nupR1/R2 alters peptide production. An important observation was the varying reduction in transcript levels of the biosynthesis genes *nupA* and *nupB* indicating that nunapeptin regulation is more complex. Furthermore, the M2D1 mutant is defective in nunapeptin synthesis and has a Tn5 insertion disruption in a novel NRPS gene cluster located far away from the *nun–nup* genes. This information points toward a regulatory cascade governing synthesis of nunamycin and nunapeptin, and potentially in addition to other secondary metabolites that may or may not play a role in the antagonism of strain In5 against plant pathogens. However, further detailed studies investigating where NunF binds combined with the generation and functional analysis of *nupR1* and *nupR2* single‐ and multiple‐site deletion mutants is required to resolve the exact role of all three regulator‐like genes in the gene regulatory network of the *nun–nup* regulon. It is also possible that NunF interacts with promoters located upstream of both *nunB1* and *nupP* which are separated by a 457‐bp region. However, no putative promoter regions were identified.

As CLPs have been shown to play important roles in antimicrobial activity and also biofilm formation and motility, a microtiter‐based biofilm assay and a swarming assay were performed to investigate whether nunamycin or nunapeptin are important for either the formation of biofilms and/or swarming by strain In5. Compared to the WT, mutant 5F5, which does not produce nunamycin, showed an 89% decrease in biofilm formation, whereas M2D1 which is defective in nunapeptin showed a 61% reduction under the conditions tested. Similarly to mutant 5F5, biofilm formation by the *nunF* mutant was reduced by 91% compared to the WT. These results indicated that In5's CLPs and, in particular, nunamycin are important for biofilm formation. Previous studies investigating the effect of CLP defective mutants in *P. fluorescens* SS101 reported a reduction in biofilm when CLP biosynthesis genes *massA*,* massB*, or *massC* are mutated (de Bruijn et al., 2008). Unlike strain SS101, CLP mutants of In5 were not mutated in swarming under the assay conditions tested.

Previous studies investigating the regulation of *Pseudomonas*‐derived CLPs, most notably in *P. syringae* pv. *syringae*, have reported three genes *salA*,* syrG*, and *syrF* to be involved in the regulation of syringomycin and syringopeptin (Lu et al., 2002, 2005; Wang et al., 2006). As discussed earlier, the organization of the strain In5 antifungal genomic island is similar to that encoding the *P. syringae* phytotoxins. However, one difference is the absence of SalA or SyrG homologs on the strain In5 antifungal genomic island. An important difference between strain In5 and *P. syringae* pv. *syringae* is that In5 is a fungal pathogen, while the latter is a plant pathogenic isolate of *Pseudomonas*, thus we would expect that regulation of antifungal compounds is different from phytotoxins. An interesting observation is the fact that In5 produces two different lipopeptides which is unusual for pseudomonads with the exception of pathogenic isolates (Raaijmakers et al., 2006). It has not yet been determined whether purified nunamycin, nunapeptin, or a combination of both peptides is phytotoxic. In *P. syringae* pv. *syringae*,* syrB* required for syringomycin production is activated in response to plant molecules (Mo & Gross, 1991). Therefore, we conducted mCherry reporter assays to determine whether *nunF* transcription is affected by environmental factors, more specifically by carbon sources indicating the presence of fungi rather than plant‐related carbon sources. Among the fungal‐associated carbon sources tested was laminarin which is a linear beta‐1,3 glucan similar to the outer parts of fungal cell walls for which we have previously observed a strong mCherry signal when In5 is grown on high concentrations of the substrate (data not shown) (Brown & Gordon, 2003; Fesel & Zuccaro, 2016; Klarzynski et al., 2000; Trouvelot et al., 2014). Oxalate and citrate are common organic acids fungi used to solubilize minerals from the environment and bacteria growing on fungal hyphae are often oxalotrophic (Bravo et al., 2013; Deveau et al., 2010; Gadd, 2007; Scheublin et al., 2010). Oxalate and citrate did not stimulate mCherry formation which may be due to the buffering capacity of the acids. The strongest signals were recorded in response to trehalose and glycerol which are typically accumulated in hyphae during stress and in particular during drought stress (Bhaganna et al., 2010; Hallsworth, 1997; Wyatt et al., 2015). In addition, glycerol has been found to be supporting bacterial growth in the fungal hyphosphere, where bacteria can ensure survival in the environment by accessing nutrients from fungal exudates (Boer, Folman, Summerbell, & Boddy, 2005; Boersma, Otten, Warmink, Nazir, & van Elsas, 2010; Nazir, Warmink, Voordes, van de Bovenkamp, & van Elsas, 2013). A strong signal was also observed for glucose which is readily mineralized by both bacteria and fungi in the rhizosphere. The stimulation of *nunF* expression in response to carbon sources indicating the presence of a fungus suggests that In5 is specialized for growth in the fungal hyphosphere. Conversely, this has been demonstrated in *P. syringae* pv. *syringae* which is specialized in plant pathogenesis, and therefore responds to plant‐associated compounds (Mo & Gross, 1991). The role of sugars as signaling molecules in plant–microbe interactions has been widely documented (Bolouri & Van den Ende, 2012; Morkunas & Ratajczak, 2014; Ortíz‐Castro, Contreras‐Cornejo, Macías‐Rodríguez, & López‐Bucio, 2009; Trouvelot et al., 2014). The ability of fungal‐associated carbohydrates to elicit an mCherry response by the In5 reporter strain indicates that they also play an important role in fungal–bacterial interactions.

Understanding the mode of action underpinning the biocontrol activity of In5 is essential for the application of this agent for the biological management of plant diseases. This study is a first step toward unraveling the regulatory network of the *nun–nup* regulon in *P. fluorescens* In5 required for nunamycin and nunapeptin synthesis which are key factors underpinning the biocontrol activity of this isolate. The NunF regulator was shown to be part of the *nun–nup* regulon and is essential for the production of both nunamycin and nunapeptin, and therefore, is also a key factor involved in the antimicrobial activity of In5. In future studies, it will be important to determine the function of *nunF* in the biocontrol activity of In5 in soil systems, and also to investigate in further detail the interplay between the additional LuxR encoding genes *nupR1* and *nupR2* and whether a more complex regulatory network involving additional genes is at play and mediating the antimicrobial activity of strain In5 through the synthesis of nunamycin and nunapeptin.

## CONFLICT OF INTEREST

The authors confirm no conflict of interest.
