# Supplementary material for: Biosynthesis of the antimicrobial cyclic lipopeptides nunamycin and nunapeptin by Pseudomonas fluorescens strain In5 is regulated by the LuxR‐type transcriptional regulator NunF
Source: Microbiologyopen. 2017 Aug 6;6(6):e00516. doi: 10.1002/mbo3.516 (PMC5727362; doi:10.1002/mbo3.516)
Supplement: Supplementary file 1 [file MBO3-6-na-s001.docx]

**LuxR** 1 MNIKNINANEKIIDKIKTCNNNKDINQCLSEIAKIIHCEYYLFAIIYPHSIIKPDVSIID
NunF 1 MNRIS------------SVRNIEN-PHIYFELGKLISSVGHEHFVANMHQLIGTSVSISL
NupR1 1 MDASP---------------------------------------------FESSDIAIHS
NupR2 1 MDTHT---------------------------------------------VKKPYLLADD

**LuxR** 61 NYPEKWRKYYDDAGLLEYDPVV-----------DY-------SKSHHSPI----------
NunF 48 VELSEWTTDDNQGSVIDIQSLGNAGLPEELSSPSSLPCSITPRQRDEHPLLQRILEVDDS
NupR1 16 NYPAEWVETYRKNALYKSDPVM-----------AN-------SAITSNPF----------
NupR2 16 IFFE-------D-AK--YQKLP-----------DFSCCLLAQDQSDKVPP----------


**LuxR** 93 ------------------NWNVFEKKTIKKESPNVIKEAQESGLITGFSFPIHTASNGFG
NunF 108 ILIHMNAPMMDAKGYQLTNATHQCNLVSGKGNRRCVITLHRPLADRDFSLSELSFLKNLS
NupR1 48 ------------------FWNEIPV----ESNTEIFEQSQEYGIQQGFSIPLHEPGRAFG
NupR2 45 ------------------KAGAQLHLTSRKNGRRYVLSVYRSHLSQGFSPQECAFLKDFS


**LuxR** 135 MLSFAHSDKD-----IYTDSLFLHASTNVPLML---------------PSLV----DNYQ
NunF 168 ETLLP----------LV--------ERHARISRQVSV--RKTGSPMARPVVAFEQTPLQR
NupR1 86 SIHLTSEDNDPDFVRIV--------RENMFIIKT-------------ISIIA----H-QY
NupR2 87 CLLLP----------MV--------EEHVAALLPSAPSRPDAHIALDEPEHGGME-TLRQ


**LuxR** 171 KINTTRKKSDSILTKREKECLAWASEGKSTWDISKILGCSERTVTFHLTNTQMKLNTTN-
NunF 208 DFNERLTLCDVALSAREKEVCLGLLTGGTVPEMAEKLCVKNSSVETYLKRAAAKLGVSG-
NupR1 120 RPIETSTESALKLTPREHEFLHWLALGKNYKEIGLIMSITERTVKFHAKQMTEKLDCINV
NupR2 128 RFADRLLESGLTLSSRETEVCVGLLAGHTAPELAEQFDLRVNTVESYLKRAAIKMGIGG-

 **LuxR** 230 R-----------------------
NunF 267 RHGLAKWMIGA-------------
NupR1 180 KQAMIKALYLN-----------LI
NupR2 187 RRSLIRWMHSVDAQPATPALRNAV

**LuxR-type DNA binding HTH domain**

**Auto-inducer binding domain**

**Supplementary Figure S1 Alignment of NunF protein to LuxR-type regulator from *Vibrio fischeri*.** Protein sequence alignment of LuxR from *V. fischeri* and NunF, NupR1 and NupR2 from *P. fluorescens* In5 *nun* – *nup* gene cluster. Blue line shows the HTH DNA binding domain of LuxR (183-230 amino acids) and green shows the auto-inducer binding domain of LuxR (24-155 amino acids).Black shading indicates identical or majority identical amino acids, grey shading indicates conserved amino acids and no shading indicates non – conserved amino acids.
