# Supplementary material for: Biosynthesis of the antimicrobial cyclic lipopeptides nunamycin and nunapeptin by Pseudomonas fluorescens strain In5 is regulated by the LuxR‐type transcriptional regulator NunF
Source: Microbiologyopen. 2017 Aug 6;6(6):e00516. doi: 10.1002/mbo3.516 (PMC5727362; doi:10.1002/mbo3.516)
Supplement: Supplementary file 2 [file MBO3-6-na-s002.docx]

**B**

**A**

SE±0.03

SE±0.02

**Growth (OD_600nm_)**

**Growth (OD_600nm_)**

**Time (hours)**

**Time (hours)**

**Supplementary Figure S2 Growth characteristics of *Pseudomonas fluorescens* In5 wild-type and strains *ΔnunF*, M2D1 and 5F5.** Growth curves in minimal medium with glucose as a sole carbon source (**A**) and complex LB medium (**B**). *Pseudomonas* sp. In5 and mutant strains were grown overnight in either LB or Defined Fusarium Medium (DFM) (Frandsen *et al.,* 2006) supplemented with 0.5% wv^-1^ glucose, shaking 200 rpm at 28ºC. Cells were washed twice with 0.9% wv^-1^ NaCl and resuspended to an OD_600nm_=0.1 and 20 µl added to a 96-well microtiter plate, 180 µl of LB or DFM 0.5% wv^-1^ glucose and growth was measured every hour for 30 hours in FLUOstar Omega Microplate Reader (BMG LABTECH, Offenburg, Germany).
