# Supplementary material for: Biosynthesis of the antimicrobial cyclic lipopeptides nunamycin and nunapeptin by Pseudomonas fluorescens strain In5 is regulated by the LuxR‐type transcriptional regulator NunF
Source: Microbiologyopen. 2017 Aug 6;6(6):e00516. doi: 10.1002/mbo3.516 (PMC5727362; doi:10.1002/mbo3.516)
Supplement: Supplementary file 3 [file MBO3-6-na-s003.docx]

**A**

******

*******

*******

**In5 *ΔnunF* M2D1 5F5 SS101**

**In5 *ΔnunF* M2D1 5F5 SS101 NC**


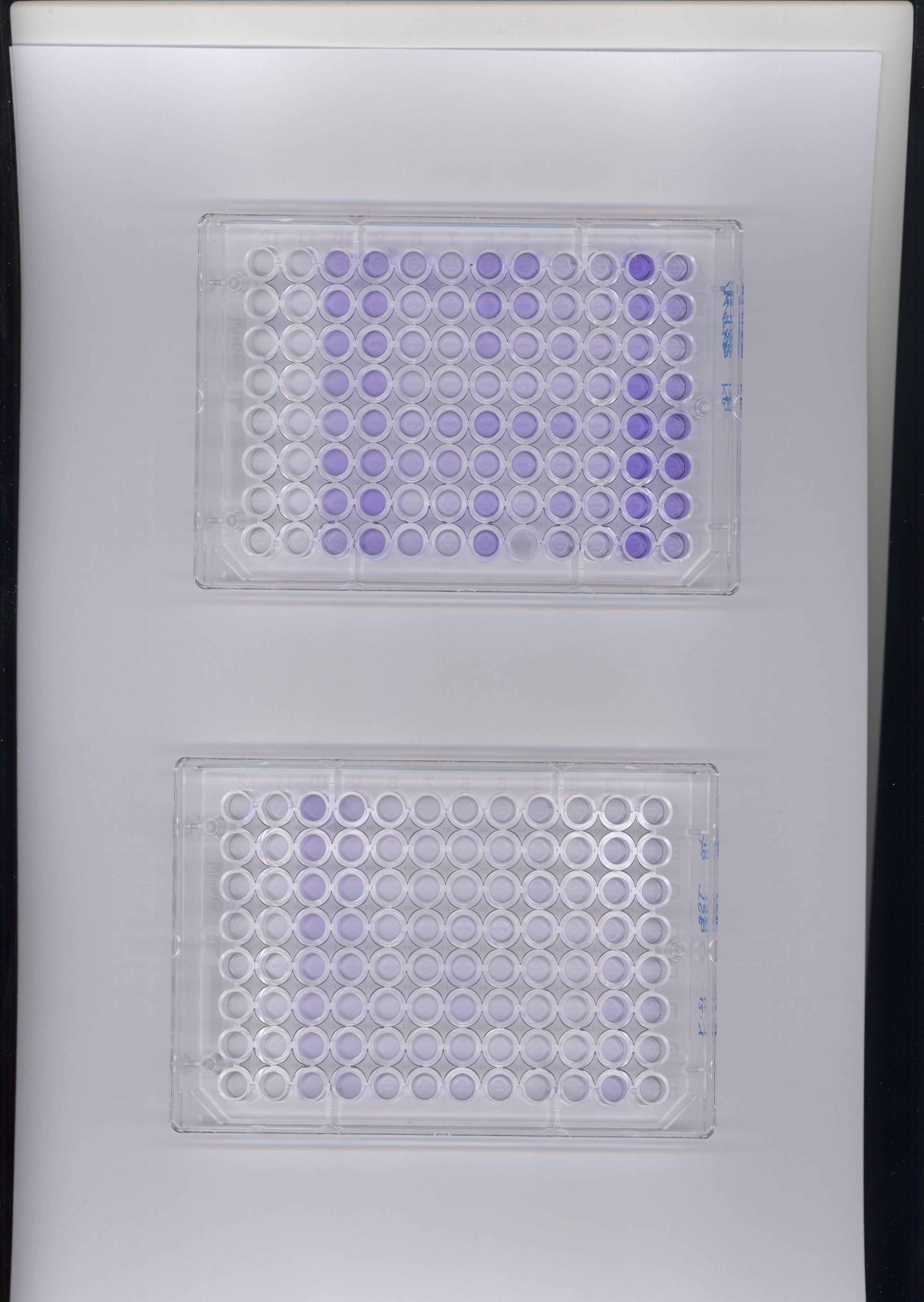


**C**

**B**

**In5 *ΔnunF* M2D1 5F5 SS101**

**Supplementary Figure S3 Biofilm formation of *Pseudomonas* *fluorescens* In5 wild-type and strains *ΔnunF*, M2D1 and 5F5.** Biofilm formation in King’s B medium (KB) broth (**A**) and planktonic cells (**B**) at 4 hours (**C**) Phenotypic appearance of biofilm formation by strains. Wells of microtiter plates were filled with 200µl of KB broth and after incubation at 25 degrees for 4 hours, cells were stained with crystal violet, wells were washed and cells attached to the walls of the microtiter plates were quantified spectrophotometrically (absorbance measured at A_595nm_). *Pseudomonas fluorescens* SS101 was used as a reference strain (**37**). Error bars represent the standard error of the means. Biofilm formation significantly different from WT strain is highlighted with an asterisk (level of significance: *<0.05, **<0.01, *** <0.001).
