# Supplementary material for: Biosynthesis of the antimicrobial cyclic lipopeptides nunamycin and nunapeptin by Pseudomonas fluorescens strain In5 is regulated by the LuxR‐type transcriptional regulator NunF
Source: Microbiologyopen. 2017 Aug 6;6(6):e00516. doi: 10.1002/mbo3.516 (PMC5727362; doi:10.1002/mbo3.516)
Supplement: Supplementary file 4 [file MBO3-6-na-s004.docx]

**A**

Tester Control

**
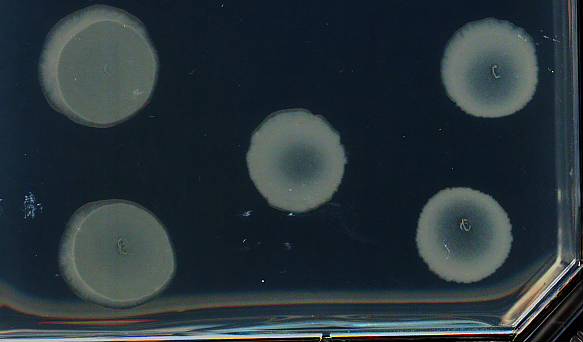

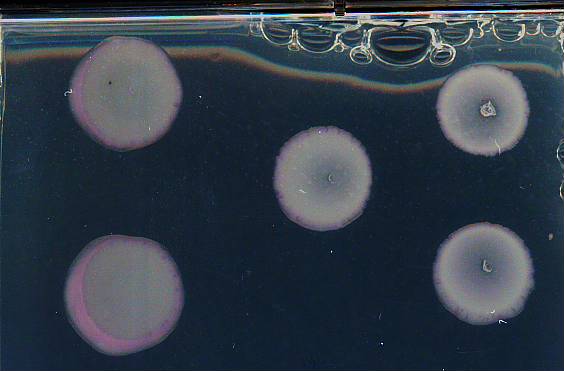
**

pSEVA237R*::*P*nunF::mCherry* pSEVA237R*::mCherry*

**B**

**C**

**Supplementary Figure S4 Analysis of the temporal response of the *nunF* promoter to rhizosphere-associated carbon sources using a *nunF*-promoter-mCherry reporter strain.** A *nunF* promoter fusion to mCherry was constructed and used as a reporter system in *P.* *fluorescens* In5 for monitoring *nunF* gene expression *in vitro*. *P.* *fluorescens* In5 reporter strain (pSEVA237R::*PnunF*::*mCherry*) and control strain (pSEVA237R::*mCherry*) were screened in a 96-well microtiter plates with varying rhizosphere-associated carbon sources. Dashed lines represent fungal-associated carbon sources and non-dashed show plant-associated carbon sources with glucose control in black. Phenotype of reporter strains spotted onto CLP-inducing medium (fifth PDA) indicates expression of *nunF* (pSEVA237R::*PnunF::mCherry*) is increased in stationary phase with no expression observed for cells growing at the edge of the colony in exponential phase (**A**), biomass of reporter strain (pSEVA237R*::mCherry*) on different carbon sources showing cells have a short lag phase entering log phase after 2 hours with most cells entering into stationary phase after 14 hours (**B**), fluorescence (mCherry signal) from the reporter strain (pSEVA237R::*mCherry*) over the control strain (empty vector; (pSEVA237R::*mCherry*) shows an increase in expression of *nunF* when cells enter stationary phase (**C**).
