# Supplementary material for: Biosynthesis of the antimicrobial cyclic lipopeptides nunamycin and nunapeptin by Pseudomonas fluorescens strain In5 is regulated by the LuxR‐type transcriptional regulator NunF
Source: Microbiologyopen. 2017 Aug 6;6(6):e00516. doi: 10.1002/mbo3.516 (PMC5727362; doi:10.1002/mbo3.516)
Supplement: Supplementary file 5 [file MBO3-6-na-s005.docx]

**Table S1 Relatedness of the NunF protein to characterised LuxR-type regulators from CLP – producing *Pseudomonads***

| Protein*^a^* | Organism*^b^* | Accession*^c^* | Length (aa) *^d^* | N-terminal domain*^e^* | C-terminal domain*^f^* | PID to NunF (%)*^g^* |
| --- | --- | --- | --- | --- | --- | --- |
| NunF | *Pseudomonas* *fluorescens* In5 | WP_054049653 | 277 | - | LUXR HTH | 100 |
| NupR1 | *P. fluorescens* In5 | WP_054050468 | 192 | AHL | LUXR HTH | 32 |
| NupR2 | *P. fluorescens* In5 | WP_054050462 | 210 | - | LUXR HTH | 41 |
| LuxR | *Vibrio fischeri* | AAQ90196 | 230 | AHL | LUXR HTH | 23 |
| SyrF | *P. syringae* pv. *syringae* | WP_016568170 | 276 | - | LUXR HTH | 57 |
| SalA | *P. syringae* pv. *syringae* | WP_016568164 | 284 | - | LUXR HTH | 29 |
| SyrG | *P. syringae* pv. *syringae* | WP_016568168 | 262 | - | LUXR HTH | 53 |
| ViscAR | *P. fluorescens* SBW25 | WP_015884801 | 264 | - | LUXR HTH | 54 |
| ViscBCR | *P. fluorescens* SBW25 | WP_043205227 | 224 | - | LUXR HTH | 33 |
| MassAR | *P. fluorescens* SS01 | ABW87979 | 264 | - | LUXR HTH | 54 |
| MassBCR | *P. fluorescens* SS01 | ABW87989 | 200 | - | LUXR HTH | 45 |

*^a^*Protein name; *^b^*Organism; *^c^*NCBI accession number; *^d^*Length of protein (aa; amino acids); *^e^*N-terminal domain based on Pfam analysis; *^f^*C-terminal domain based on Pfam analysis; *^g^*PID; percentage identity at protein level to NunF protein from *P. fluorescens* In5.
