# Supplementary material for: Biosynthesis of the antimicrobial cyclic lipopeptides nunamycin and nunapeptin by Pseudomonas fluorescens strain In5 is regulated by the LuxR‐type transcriptional regulator NunF
Source: Microbiologyopen. 2017 Aug 6;6(6):e00516. doi: 10.1002/mbo3.516 (PMC5727362; doi:10.1002/mbo3.516)
Supplement: Supplementary file 6 [file MBO3-6-na-s006.docx]

**Supplementary Table S2: Swarming motility of *Pseudomonas* *fluorescens* In5 and mutant strains**

| **Strain** | **Agar (%)** | **Swarming Phenotype** | **Colony (diameter mm)** |
| --- | --- | --- | --- |
| WT | 0.25 | featureless | 35.0±0.0 |
| WT | 0.6 | non - swarming | - |
| *ΔnunF* | 0.25 | featureless | 30.0±1.0 |
| *ΔnunF* | 0.6 | non - swarming | - |
| M2D1 | 0.25 | featureless | 36.0±1.0 |
| M2D1 | 0.6 | non - swarming | - |
| 5F5 | 0.25 | featureless | 36.7±0.9 |
| 5F5 | 0.6 | non - swarming | - |
| SS101 | 0.25 | dendritic | - |
| SS101 | 0.6 | dendritic | - |

Strains tested in triplicate for each treatment are *P*. *fluorescens* In5 wildtype and mutant strains *ΔnunF*, 5F5 and M2D1 and reference strain

*P. fluorescens* SS101 cultivated for 48 hours at 25ºC
