# Supplementary material for: Biosynthesis of the antimicrobial cyclic lipopeptides nunamycin and nunapeptin by Pseudomonas fluorescens strain In5 is regulated by the LuxR‐type transcriptional regulator NunF
Source: Microbiologyopen. 2017 Aug 6;6(6):e00516. doi: 10.1002/mbo3.516 (PMC5727362; doi:10.1002/mbo3.516)
Supplement: Supplementary file 7 [file MBO3-6-na-s007.docx]

**Supplementary Table S3 Standard error of the mean of biomass values from *P. fluorescens* In5 growth of different carbon sources**

| **Carbon source** | **24H** | **30H** | **48H** |
| --- | --- | --- | --- |
| Raffinose | 0.00367 | 0.00283 | 0.00933 |
| Fucose | 0.00733 | 0.01217 | 0.00017 |
| Trehalose | 0.00533 | 0.001 | 0.00267 |
| Glycerol | 0.029 | 0.022 | 0.006 |
| Cellobiose | 0.00133 | 0.00417 | 0.00117 |
| Laminarin | 0.002 | 0.0035 | 0.0015 |
| Inisitol | 0.00917 | 0.00967 | 0.001 |
| Arabinose | 0.005 | 0.00217 | 0.00767 |
| Oxalate | 0.00133 | 0.008 | 0.00633 |
| Glucose | 0.00117 | 0.0085 | 0.0035 |
| Laminarin-triose | 0.0165 | 0.0165 | 0.00583 |
| Citrate | 0.0015 | 0.00033 | 0.00383 |
